# Cost-utility analysis of atezolizumab with bevacizumab in untreated unresectable or advanced hepatocellular carcinoma in France

**DOI:** 10.1371/journal.pone.0280442

**Published:** 2023-01-18

**Authors:** Loïg Gaugain, Hélène Cawston, Coline Dubois de Gennes, Javier Sanchez Alvares, Pierre Nahon, Benjamin Mazaleyrat, Clément Le Dissez

**Affiliations:** 1 Amaris Consulting, Montréal, Canada; 2 Amaris Consulting, Paris, France; 3 Amaris Consulting, London, United-Kingdom; 4 Roche SAS, Boulogne-Billancourt, France; 5 AP-HP, Liver Unit, Bobigny, France; 6 Université Sorbonne Paris Nord, Bobigny, France; IRCCS Giovanni Paolo II Cancer Hospital, ITALY

## Abstract

**Background and aims:**

The IMbrave150 clinical trial assessed the efficacy and safety of atezolizumab in combination with bevacizumab (ATZ+BVA) versus sorafenib in adults with advanced/unresectable hepatocellular carcinoma, who have not received prior systemic treatment. Our aim was to assess the cost-effectiveness of ATZ+BVA versus sorafenib in France based on an updated prices and considering French National real-world data, to confirm the initial recommendations from the Heath Technology Assessment submission published in 2021, and provide additional visibility to decision-makers reflecting current clinical practice.

**Methods:**

A partition survival model was developed to project clinical outcomes, quality of life, and costs of patients with HCC treated with ATZ+BVA versus sorafenib over a lifetime horizon. Survival outcomes were extrapolated via parametric functions for both treatment strategies. Quality of life (EQ-5D-5L, French tariffs) were sourced from IMbrave150. The Guyot method was considered as a scenario analysis by integrating retrospective real-world data extracted from the French Health Insurance Database to refine long term survival extrapolations.

**Results:**

In the reference case, ATZ+BVA was associated with 0.61 additional Quality Adjusted Life Years (QALYs) compared to sorafenib (1.95 vs 1.35), and an incremental cost of €92,704. The incremental cost-utility ratio (ICUR) was 152,974 €/QALY gained. Adjusting the survival curves with French external evidence led to a 14% ICUR reduction (131,163 €/QALY).

**Conclusions:**

ATZ+BVA is a cost-effective strategy based on the range recently published for the value of a QALY in France and offers better chances of survival to patients.

## Introduction

Liver cancer incidence has increased worldwide in recent years. In France, primary liver cancer is the second most frequent digestive cancer in men and third most frequent cancer in women [1] with 10,580 total cases reported in 2018 [2]. Hepatocellular carcinoma (HCC) represents 90% of all primary liver cases in France [3]. Surveillance programs aim to detect HCC at early stages in patients with advanced chronic liver disease [4, 5]. However, globally, 25% to 70% of patients are diagnosed with advanced stages of HCC, meaning that the cancer has progressed after being asymptomatic for a long time [6].

HCC is associated with poor survival and represents the fourth and seventh cause of cancer deaths in France in men and women, respectively. It amounted to a total of 8,697 deaths recorded in 2018 [1]. Prior to the implementation of immunotherapy, a French registry study conducted in patients diagnosed with HCC between 2009 and 2012 reported a median survival of 9.4 months (for all disease stages) from diagnosis and a five-year survival rate of 9.6% [7]. More recently, a French retrospective study based on 2019 data (ATHENOR) estimated the survival rates of HCC patients at all stages treated with sorafenib at 37.5% and 17.9% at one and two years, respectively [8].

Immunotherapy and in particular immune checkpoint inhibitors (ICIs) have been introduced to the therapeutic landscape of several haematological and solid tumours [9, 10]. ICIs monotherapies have been assessed in randomised trials settings in first- and second-line HCC treatment but failed to show significant improvements in survival outcomes [11]. However, ICIs combinations with other agents strategies, such as anti PD-L1+antiangiogenic molecular targeted therapy or anti CTLA-4 have shown promising results in clinical trials [9, 11]. In October 2020, a marketing authorization for the combination ATZ+BVA was granted by the European Medicines Agency (EMA) for the treatment of adult patients with advanced or unresectable hepatocellular carcinoma (HCC) who have not received prior systemic therapy [12]. ATZ+BVA has been available in France since July 2020 through an early access program (*Autorisation Temporaire d’Utilisation [ATU]*) and is the first treatment approved for reimbursement in the first-line setting since sorafenib in 2008 [13]. Atezolizumab, in combination of bevacizumab is currently the only treatment approved by the French health technology assessment body, *Haute Autorité de Santé* (HAS), for first-line treatment of HCC patients with preserved liver function (Child Pugh A) [13], following sorafenib in 2008. The efficacy and safety of ATZ+BVA versus sorafenib as first line treatment for patients with locally advanced or metastatic and/or unresectable HCC was assessed in a phase III, randomised, open-label clinical trial (IMbrave150). The trial showed that ATZ+BVA led to statistically significant and clinically meaningful prolonged progression-free survival (PFS) and overall survival (OS) compared to sorafenib [14].

These new treatments, however, represent an important cost burden for National Health Systems. Therefore, the cost of ATZ+BVA needs to be compared to the current cost of treating HCC in France, alongside the survival benefits it could offer to patients. In 2021, the HAS published an evaluation of the cost-effectiveness of ATZ+BVA versus sorafenib for first-line treatment of adults with advanced/unresectable HCC in France, based on the price initially requested by the manufacturer [15]. Although no official threshold of maximum willingness-to-pay for an additional Quality Adjusted Life Year (QALY) has been published in French guidelines, Téhard et al. estimated that the value of a QALY in France ranges between €147,093 and €201,398 [16]. In the 2021 technology appraisal, the HAS estimated the ICUR between ATZ+BVA versus sorafenib to be at 144,156 €/QALY, falling below the reported maximum willingness-to-pay [15].

The objective of this study was to offer more visibility for decision-makers by updating the analysis presented in the HAS opinion with the most recent generic price for sorafenib, while also considering recent clinical guidance (ESMO guidelines Committee 2021), whereby sorafenib can also be considered in patients who progressed with ATZ+BVA. Given the limited patient follow-up in the pivotal study IMbrave150, we investigated the use of the Guyot Method [17] considering external real-world data from a retrospective database in France to adjust survival extrapolations and strengthen the robustness of results as part of a scenario analysis.

## Methods

### Population simulated in the analysis

The population simulated corresponds to the population of the IMbrave150 clinical trial and is consistent with the population eligible for reimbursement in France [15]. We simulated patients with advanced or unresectable HCC who have not received prior systemic treatment, have a preserved liver function (Child-Pugh A), are ineligible to or have progressed on locoregional treatments, and have a performance status (Eastern Cooperative Oncology Group [ECOG] score) between 0 and 1. Baseline characteristics (mean age, sex ratio, body weight, height and body surface area) were extracted from the IMbrave150 trial.

### Structuring choices

In our analysis, the number of life years (LY) gained for each treatment strategy (ATZ+BVA or sorafenib) was weighted by patients’ quality of life. The cost-effectiveness of ATZ+BVA versus sorafenib was estimated by dividing the incremental costs by the incremental QALYs.

We used a partitioned survival model to estimate costs and clinical outcomes of ATZ+BVA versus sorafenib over a lifetime horizon, estimated at 15 years given the disease evolution and epidemiological data evidence [13]. Within a partitioned survival model, health states are based on the partitioning of the proportion of patients alive into on treatment/ progression free and off treatment/ progression at discrete time points. This structure is typically utilized in pharmacoeconomic analyses related to oncology [18]. The model explored three health states (pre-progression, post-progression, and death) as well as the proportion of patients on or off treatment, derived from OS, PFS, and time to off treatment (TTOT) curves. The OS curve describes time from model entry to death and is used to directly determine the proportion of patients alive (and dead) over time. The PFS curve describes the time from model entry to exiting the progression-free state via progression or death and provides the proportion of patients that are progression-free over time. For the progressed health state, state membership is derived as the difference between the OS and the PFS curve at each point, because this represents the proportion of patients who are alive but not progression free. The model inputs were based on the results of IMbrave150.

Costs and clinical outcomes were discounted at 2.5% as recommended in the French methodological guidelines of HAS in 2020 [19].

### Efficacy and safety data from IMbrave150

Most clinical data used in the cost-utility analysis was estimated from the individual patient-level data from the IMbrave150 clinical trial. The first interim analysis of IMbrave150, based on the August 2019 cut-off, showed that ATZ+BVA provides a clinically meaningful and statistically significant benefit compared to sorafenib in terms of OS and PFS [14]. Moreover, IMbrave150 showed the longest survival among all phase III studies conducted in first-line treatment of advanced/unresectable HCC. A descriptive updated analysis in August 2020 added another 12 months of follow-up (median follow-up of 15.6 months). Median OS was 19.2 months with ATZ+BVA versus 13.4 months with sorafenib (HR, 0.66 [95% Confidence Interval CI, 0.52, 0.85]; P = 0.0009) [20].

IMbrave150 safety data indicated that grade 3 and 4 adverse events occurred in 56.5% in patients treated with ATZ+BVA compared to 55.1% in those treated with sorafenib. Most common grade 3–4 event in ATZ+BVA arm was hypertension, a finding consistent with the known bevacizumab safety profile [14]. Grade 5 events occurred in 4.6% of patients treated with ATZ+BVA versus 5.8% in those treated with sorafenib. Other high-grade toxic effects were not observed frequently.

### Integration of survival data from the clinical trial

Survival outcomes came from the August 2020 analysis of the IMbrave150 trial for both ATZ+BVA and sorafenib and were extrapolated over 15 years. A 15-year time horizon was used as survival data in the ATHENOR study indicated a survival rate below 2% after 106 months. Based on statistical fit (through the Akaike Information Criterion [AIC] and Bayesian Information Criterion [BIC] metrics) and clinical plausibility, OS, PFS and TTOT curves were fitted via independent exponential parametric distributions for both arms. As part of a scenario analysis, the OS was estimated with the integration of external evidence through Guyot’s method. This statistical method allows for the inclusion of real-life long-term data in the estimation of the parameters of the distributions, under certain assumptions. As such, this method enriches and refines the extrapolation of the survival curves to account for all available evidence. The adjustment of the long-term predictions is done through a forced convergence of the conditional survival of the control arm to the conditional survival of the external data (i.e., the ATHENOR study). Such an approach allows the extrapolation from a parametric independent model to consider external evidence, leading to predictions that are more robust and clinically plausible. The survival parameters are then estimated using information from both the clinical trial and the real-life long-term data.

### Integration of survival using additional ATHENOR data (scenario analysis)

ATHENOR is a retrospective observational study based on the French database SNDS (*Système National des Données de Santé*), which includes claims data for over 65 million individuals covering 99% of the total population. ATHENOR aimed to describe the characteristics, management, OS, duration of treatment, and level of resource use of advanced HCC patients treated with first-line sorafenib [8]. The study included 17,680 patients that had received at least one dose of sorafenib between 2009 and 2018 [21]. Patients treated with sorafenib were predominantly male (87.6%) and had a median age of 68 years. Main causes of chronic liver diseases were alcoholic liver disease (55%), hepatitis C (20%), hepatitis B (7%), and Non-Alcoholic Steatohepatitis [NASH]/Non-Alcoholic Fatty Liver Disease [NAFLD] (7%). Patients could have several causes of liver disease present at the same time. A median survival of 8.4 months (95% CI [8.2; 8.7]) from initiation of sorafenib was reported.

**Fig 1:**
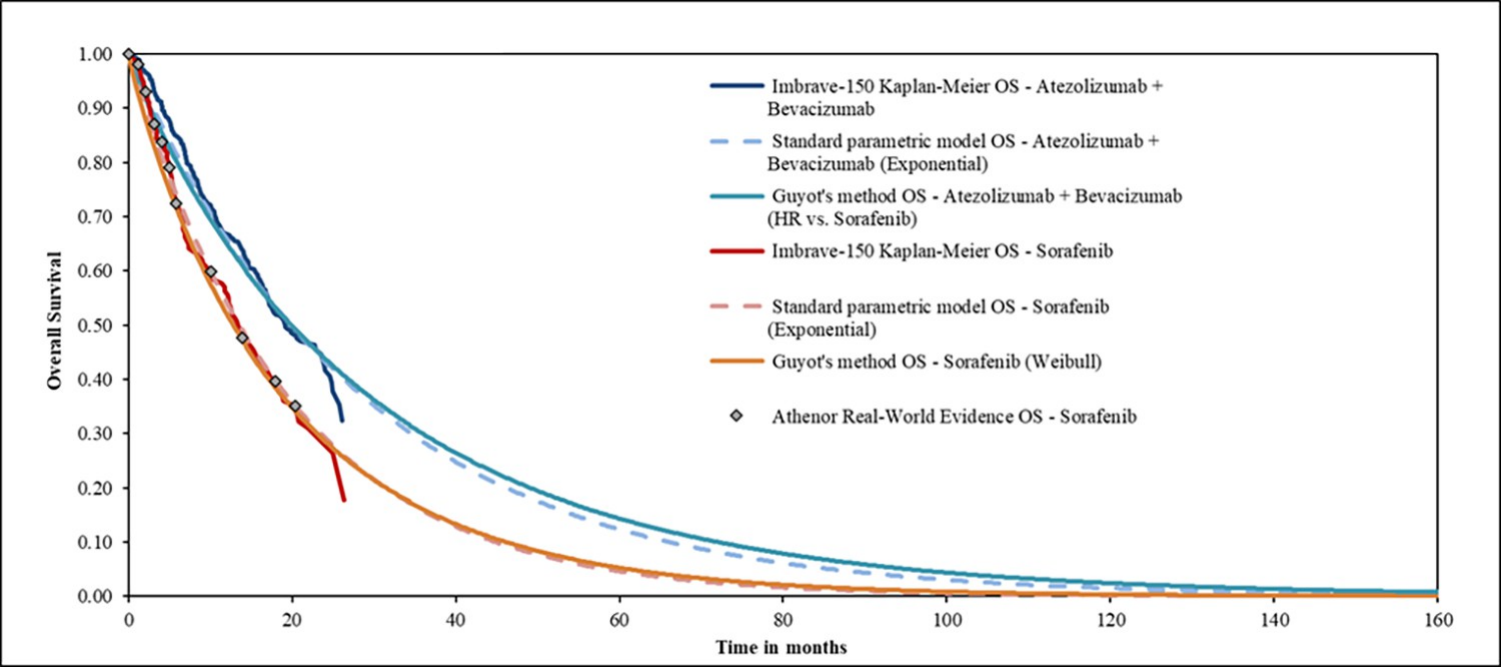
OS extrapolations for ATZ+BVA and sorafenib with the Guyot’s method.

Standard extrapolations solely rely on individual patient-level data from the pivotal trial with external evidence used for validation purposes only. Innovative statistical models, such as Guyot’s method, can adjust survival models based on external data in a Bayesian framework [17]. Guyot’s underlying assumption is that over a long-term horizon, the annual conditional survival (i.e., the probability of surviving additional years given that the patient has already survived a certain number of years after treatment initiation) in the clinical trial control arm is likely the same as that observed in observational studies. We applied this method to estimate overall survival in the sorafenib arm in the scenario analysis. A primary conditional survival constraint was included based on ATHENOR after 20 months. This time point corresponds to 70% of the follow-up in IMbrave150, after which the Kaplan-Meier curve changed considerably in the sorafenib arm due to censorship. This might have led to a more pessimistic survival in the control arm without further adjustment as the survival decreases after 20 months with a low number of patients at risk. The Weibull distribution was selected amongst the standard parametric distributions based on Deviance Information Criterion [DIC] criteria and clinical plausibility. A second constraint associated with Guyot’s method is that the conditional survival associated with the control arm must remain lower than the one observed in the general population, which was based on French data [17]. Overall survival in the ATZ+BVA arm was derived by applying the hazard ratio (HR = 0.66 [95%CI: 0.52, 0.85]) from IMbrave150 to the sorafenib arm as the proportional hazard assumption was supported. The extrapolated OS for ATZ+BVA was adjusted to ensure that at each cycle the probability of survival did not exceed that of the French general population. The impact of adjusting OS through French external evidence is represented in Fig 1. This stratified HR was retrieved from the OS main dependent analysis undertaken in the clinical cut-off date of August 31st (2020) on the Intention-to-Treat population.

### Quality of life

Quality of life was assessed in IMbrave150 via the EQ-5D-5L questionnaire. The questionnaire was administered at the first day of every cycle until the medical visit leading to the treatment discontinuation. The questionnaire was completed by the patient after disease progression or treatment discontinuation every three months for a year if patients maintained their consent. In total, 4,712 EQ-5D-5L questionnaires were collected in the IMbrave150 trial. Linear mixed effects models were employed to account for repeated measures among patients. The weighting matrix described in Andrade et al. 2020 [22] was used to generate utility scores specific to the French population. Utility scores were estimated by health state, which reflected utility before and after progression (see Table 1). Utilities were analysed using a mixed model adjusting for centralised baseline utilities using the patient identifier variable as a random intercept.

**Table 1:** Utility scores per health state (EQ-5D-5l analysis from IMbrave150).

	Mean	(SE)	Lower bound (CI 95%)	Higher bound (CI 95%)
**Utility score pre-progression**	0.8933	0.0059	0.8842	0.9030
**Utility score after progression**	0.8634	0.0064	0.8534	0.8738

CI: Confidence Interval; SE: Standard Error.

Additional disutility decrements were considered to reflect a reduction of quality of life in patients experiencing adverse events (observed in IMbrave150 clinical trial). Grade 3 and 4 adverse events that occurred in the clinical trial with a frequency higher than 1% were included as one-off event at first cycle. Utility decrements came from various sources in the literature, and when data were missing, higher decrements found in the literature were applied. Patients’ quality of life throughout the trial rapidly dropped as patients approached death. In an additional scenario, a mean utility score of 0.6109 (Standard Error [SE]: 0.0203) was considered to account for this decrease in the five weeks before death.

### Costs and medical resource use

Costs were estimated from a collective perspective meaning that all resources directly involved in the production of care of patients with HCC were considered. All costs were inflated to €2019 based on the health and personal care component of the Consumer Price Index [23]. Unit costs were associated with specific levels of medical resource use, based on French or European clinical practice. Where information was missing, experts’ opinion was collected via questionnaires to French Key Opinion Leaders (KOL). Treatment acquisition costs were identified via the national database of drug prices in France (*Base des Médicaments et information Tarifaires*) with dosing schedules based on the summary of product characteristics [24]. The cost of administration of ATZ+BVA was estimated from the 2017 National Study of Costs in France (*Etude Nationale des Coûts [ENC]*) considering a weighted average between the private and the public sector [25]. Additional transport costs were accounted for in case of transport to the hospital for administration, management of adverse events, or follow-up. Follow-up costs included monitoring, biological examinations, and physician visits. Costs of all-cause grade 3 to 4 adverse events were estimated through the 2017 ENC and included in the analysis. Second-line post-progression treatment costs for regorafenib, cabozantinib, and sorafenib (after ATZ+BVA only) were considered. The analysis also accounted for end of life costs for all patients. All costs and resource use considered are presented in Table 2.

**Table 2:** Costs and level of resource use.

Cost item	Unit cost €	Weekly cost €	Reference
Specific to ATZ+BVA			
**Acquisition cost of Atezolizumab**	3,569.52 per cycle (3 weeks)	1,189.84	BdM_IT (French National Price Database)
**Acquisition cost of Bevacizumab**	1,746.57 per cycle (3 weeks)	582.19	
**Administration of ATZ+BVA**	483.50	161.17	ENC 2017 (National Cost Study)
**Follow-up costs for ATZ+BVA**	-	59.00	
**Handling AE for ATZ+BVA (2**^**nd**^ **cut off)**		31.10	IMbrave150 (2^nd^ cut-off)
**Total costs of 2L treatments (acquisition, administration, and AE related to regorafenib, cabozantinib and sorafenib) (2**^**nd**^ **cut off)**	11,729.34	-	BdM_IT (French National Price Database)
Specific to sorafenib			
**Acquisition of sorafenib**	1,280.10 for 112 pills	320.03	BdM_IT (French National Price Database)
**Dispensing fee**	1.53	0.51	Ameli website (French National Payer)
**Follow up costs for sorafenib**	NA	32.88	ENC 2017 (National Cost Study)
**Handling AE for sorafenib (2**^**nd**^ **cut off)**	-	41.65	IMbrave-150 (2^nd^ cut off)
**Total costs of 2L treatments (acquisition, administration, and AE related to regorafenib and cabozantinib) (2**^**nd**^ **cut off)**	22,646.62	-	BdM_IT (French National Price Database)
Common costs for both strategies			
Transport cost (included in administration cost for ATZ+BVA)	52.42 for return trip	NA	Report from Cour des Comptes 2019
			Report from IRDES 2011
Follow up costs post progression	NA	32.88	ENC 2017 (National Cost Study)
End of life cost	6,357.58	NA	

ATZ+BVA: Atezolizumab + Bevacizumab; BdM_IT: Base des médicaments et des informations tarifaires; IRDES: Institut de recherche et documentation en économie de la santé; AE : Adverse events; NA: not aplicable.

### Exploration of uncertainty

Sensitivity and scenario analyses were conducted to explore the impact of uncertainty and assumptions on model outcomes. Deterministic one-way analyses were developed to identify the influence of individual parameters. When confidence intervals were not available, a range of ±10% of the base-case values was considered. Probabilistic multivariate sensitivity analyses were run based on 1,000 iterations varying all considered parameters based on sampling distributions. Costs were assigned gamma distributions. Utility values, probabilities, or proportions were assigned beta distributions assuming the standard deviation of 10% from mean values when robust data were not available.

## Results

### Base case analysis

Over a 15-year time horizon, patients in the ATZ+BVA were predicted to live for 2.26 discounted years, and patients in the sorafenib arm were predicted to live for 1.57 discounted years. In the ATZ+BVA arm, longer survival was observed both pre and post progression (+0.35 years and +0.34 years, respectively). Patients treated with ATZ+BVA and sorafenib were associated with 1.95 and 1.34 QALYs, respectively. Incremental discounted costs incurred by patients treated with ATZ+BVA amounted to €120,923, largely driven by the acquisition cost of ATZ+BVA (79% of total costs). Incremental costs and benefits of ATZ+BVA vs. sorafenib lead to an incremental cost per life years gained ratio of 133,809 €/LY and cost per QALY gained ratio of 152,974 €/QALY. Base case results are detailed in Table 3.

**Table 3:** Results of base case analysis.

	ATZ+BVA	Sorafenib
** **LYs	2.26	1.57
LYs–pre-progression	0.95	0.61
LYs–post-progression	1.30	0.96
QALYs	1.95	1.35
QALYs–pre-progression	0.83	0.52
QALYs–post-progression	1.13	0.83
** **Total costs	€120,923	€28,220
**Acquisition costs**	€95,013	€6,887
**Administration costs**	€8,857	€9
**Follow-up costs**	€5,175	€2,686
**Cost of adverse events**	€1,677	€896
**Cost of post-progression active treatment**	€4,206	€11,629
**End of life costs**	€5,995	€6,112
** **Incremental results (ATZ+BVA)		
** Incremental LYs**	0.69	-
** Incremental QALYs**	0.61	-
** Incremental costs**	€92,704	-
** Incremental Cost per QALY gained**	**152,974 €/QALY**	-

LY: Life years, QALY: Quality Adjusted Life Years, ATZ: atezolizumab, BVA: bevacizumab.

### Scenario analysis

The OS adjustment through the Guyot method led to 2.37 predicted discounted life years for ATZ+BVA and 1.56 for sorafenib overall with higher gains in post progression survival (+0.46 years compared to +0.35 years in pre progression). Incremental costs and benefits led to an incremental cost per life year ratio of 114,514 €/LY and cost per QALY gained ratio of 131,163 €/QALY. This scenario analysis led to a 14% decrease in the ICUR, indicating that the use of trial data alone was a conservative approach. The inclusion of utility scores per health states accounting for the utility drop in the five weeks before death led the ICUR to decrease by approximately 12%, which corresponds to 135,036 €/QALY.

### Exploration of uncertainty

**Fig 2:**
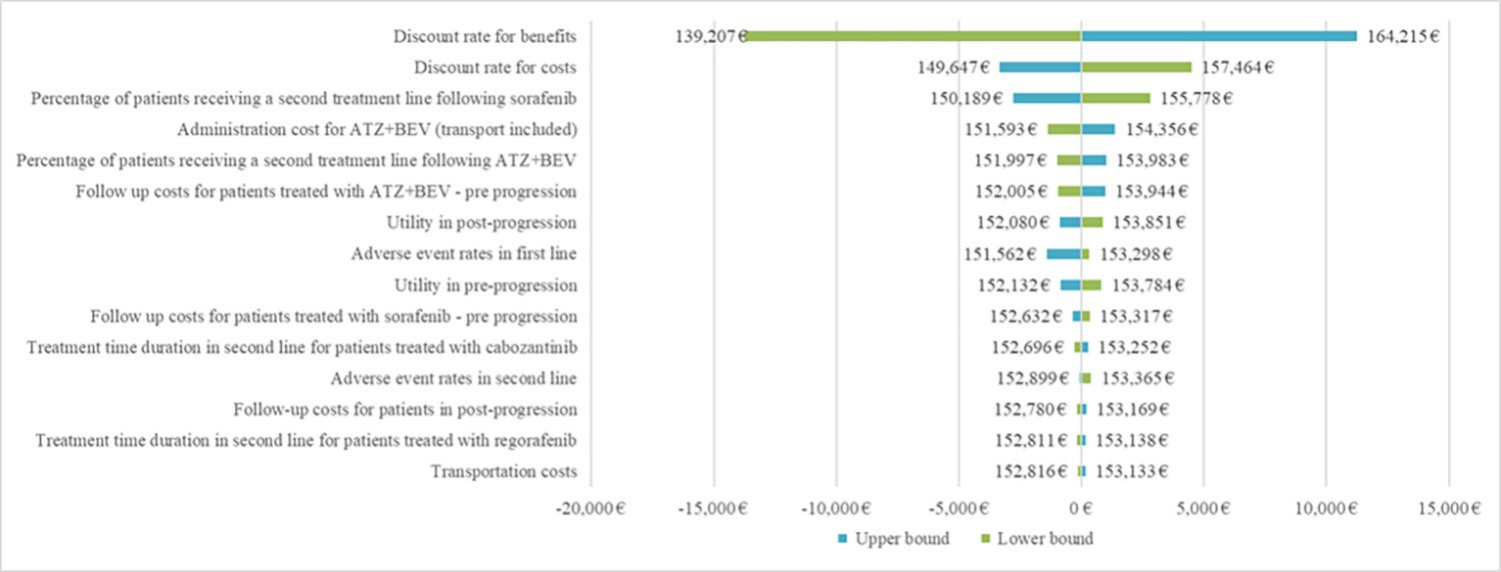
Deterministic sensitivity analysis.

The deterministic sensitivity analysis showed that discount rates for both benefits and costs, as well as the proportion of patients receiving a treatment after progression, had the largest impact on the ICUR (see Fig 2).

**Fig 3:**
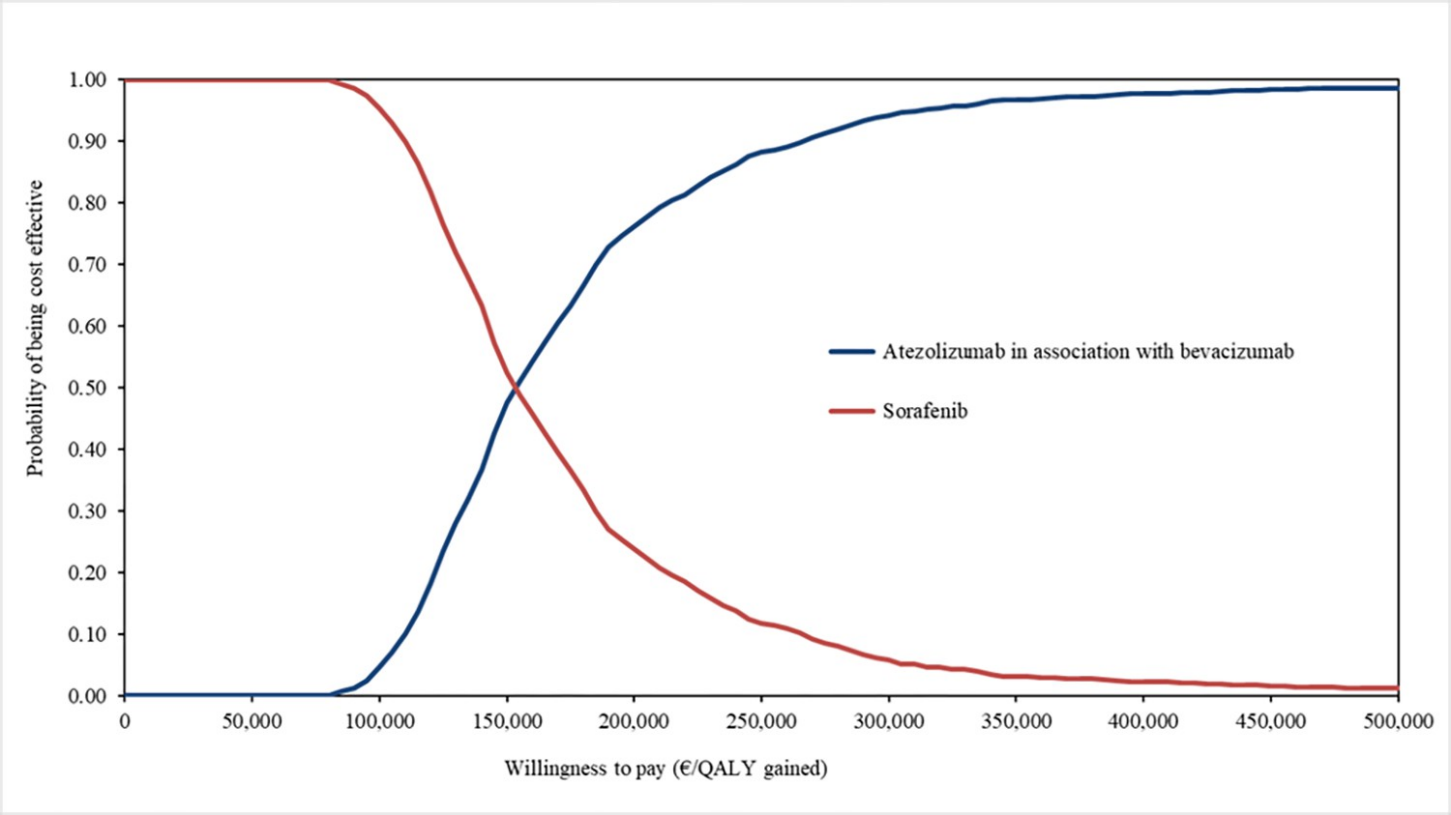
Cost-effectiveness acceptability curves from probabilistic sensitivity analysis.

Based on the 1,000 iterations of the probabilistic sensitivity analysis, ATZ+BVA had a 24% probability of being cost-effective for a willingness to pay of €125,000 per QALY (Fig 3).

## Discussion

This study is the first cost-utility analysis of first-line treatment for advanced HCC in France. The analysis used a classic partitioned survival model structure, which has been previously used by European HTA bodies in past assessments of interventions for HCC [18]. Most data in the model (i.e., efficacy, adverse events, quality of life) came from IMbrave150, a relevant and robust phase III, randomised, clinical trial which directly compared ATZ+BVA and sorafenib. Good methodological consistency was assured throughout the analysis since it did not rely on any efficacy data from indirect comparisons. Furthermore, most clinical and cost assumptions were confirmed with key opinion leaders, and the external validity of the model was explored with real-world evidence.

Our study has several limitations. First, survival extrapolations are a source of uncertainty. Standard parametric models were considered for model selection, while more flexible models (splines and other nonparametric regression models) could have been considered. However, conservative approaches were used throughout, and Guyot’s method was considered in a scenario analysis. This provided long-term survival extrapolations which reflected observed survival in the French retrospective database. A second limitation, inherent to Guyot’s method and acknowledged by its authors, is that the approach relies on the assumption that relative conditional survival provides an accurate way of extrapolating data from the trial. As this paper constitutes the first application known to the authors of the Guyot’s method in this indication, it is difficult to conclude over the relationship between relative conditional survival and long-term extrapolations in HCC. In addition, overall survival of the ATZ+BVA arm was reproduced with the hazard ratio from IMbrave150. Although the Guyot’s method added a constraint on the survival curve through the conditional hazard rate, the long-term validity of the proportional hazard assumption cannot be verified. The degree of reliance on the use of the French retrospective database to adjust the treatment effect on OS is difficult to assess. There is little evidence in the literature regarding the impact of the Guyot’s method over the conclusions of a cost-utility analysis. Our analysis demonstrates the benefits of investigating the impact of external data on long term extrapolations through the Bayesian Guyot Method, which is not included as a standard practice in the National Institute for Clinical Excellence Decision Support Unit (NICE DSU) technical support document [26] and rarely presented in the context of HTA appraisals.

Limited data on quality of life was available in the literature in this specific indication at the time of the analysis. Considering trial-based utility scores per health state is a broadly accepted method in pharmacoeconomic analyses in oncology, which is why this was preferred as a base case. However, patients’ quality of life can decline progressively and a finer method considering additional time to death utility showed a decrease of approximately 30% in the model’s results. Most uncertainties in the model were addressed by using conservative approaches. For example, there is currently no clear consensus on the level of medical resource utilisation in patients with advanced HCC treated with ATZ+BVA. Therefore, the model assumes a higher frequency of follow-up visits for patients with ATZ+BVA versus patients treated by sorafenib only, thereby increasing the total cost of ATZ+BVA arm. Although there is a chance patients treated with sorafenib may have to go to the hospital more often because of adverse events.

The integration of post-progression treatments, such as cabozantinib or regorafenib, was validated by clinicians, and reflect the current clinical practice in France. In the IMbrave150 clinical trial, around 16% of patients in the sorafenib arm had received nivolumab (versus 3% in the ATZ+BVA arm), which is a treatment not accounted for in the model as it does not have a marketing authorization in France in this indication. Consequently, no costs incurred by the use of nivolumab were considered, whereas its potential clinical benefits may be reflected in the incremental benefit, and therefore, the ICER. In the IMbrave150 clinical trial, most patients had a viral aetiology for HCC. However, as suggested in the ATHENOR study, the main aetiology for French patients with HCC is alcohol related. There is a need to explore cost-effectiveness in patients with non-viral aetiology specifically. Other sub-groups exploring the cost-effectiveness of patients according to their ECOG status or the Barcelona Clinical Liver Cancer (BCLC) stage would be also interesting to explore.

The arrival of innovative cancer immunotherapies on the French market can have a sizable impact on French payers and on the sustainability of the national healthcare system. Nevertheless, results from this cost-utility model are likely overestimated in this indication. This analysis was primarily based on a conservative approach and was conducted using public list prices for ATZ+BVA, which does not account for confidential rebate from the Economic Comity of Health Products (CEPS) in France. Additionally, the arrival of BVA biosimilars will drive the prices down in the hospital setting. According to the CEPS 2020 report activity, pricing and regulations agreements state initial discount rates of 30% on biosimilars and their biologic reference drugs in the hospital setting [27]. Considering lower prices for both ATZ and BVA will result in considerable reductions in the incremental ratio. However, sorafenib is not likely to undergo future confidential rebates as it has been on the market for over ten years and has a generic price today.

In 2020, two cost-utility analyses were published based on the IMbrave150 clinical trial. These studies were assessed by the National Institute for Health and Care Excellence (NICE) [28] and the Canadian Agency for Drugs and Technologies in Health (CADTH) [29]. Both analyses used a partitioned survival model and concluded that ATZ+BVA was cost-effective versus sorafenib and lenvatinib by extrapolating costs and benefits over a 20- and 10-years time horizon, respectively, However, our ICUR point estimates were substantially higher than NICE and 33% lower than CADTH. These differences can be explained by differences in country-specific inputs, such as weighting matrices for utilities, or specific costs incurred in each country, differences in local clinical guidance, the chosen perspective, and discount rates (3.5% in the NICE appraisal versus 2.5% in our analysis). Finally, our analysis was based on the generic price for sorafenib which means lower total costs for sorafenib, hence a higher incremental ratio.

Finally, these analyses confirmed that atezolizumab in combination with bevacizumab can be considered cost-effective as a first line option for patients with untreated unresectable or advanced hepatocellular carcinoma in the French context and confirm the critical assessment made by the HAS. Although the combination is associated with higher initial costs, it is expected to generate savings after disease progression. The incorporation of real-world evidence though the Guyot method strengthens the robustness of the survival extrapolations, reflecting the potential benefits associated with the combination in French patients.

## Supporting information

S1 Table Parameters included in the cost-utility analysis. (DOCX)
