# Supplementary material for: Cost-utility analysis of atezolizumab with bevacizumab in untreated unresectable or advanced hepatocellular carcinoma in France
Source: PLoS One. 2023 Jan 18;18(1):e0280442. doi: 10.1371/journal.pone.0280442 (PMC9847974; doi:10.1371/journal.pone.0280442)
Supplement: S1 Table — (DOCX) [file pone.0280442.s001.docx]

## Supporting Information file – Parameters included in the cost-utility analysis

| Parameters | Values |
| --- | --- |
| Annual Discount rate (costs) | 2.5% |
| Annual Discount rate (effects) | 2.5% |
| Time horizon of the analysis (years) | 15 |
| Baseline age | 63 |
| Baseline body weight | 71.74 |
| Baseline height | 168.47 |
| Baseline BSA | 1.82 |
| Proportion of patients PD-L1 (for testing cost) | 0.0% |
| Proportion of male patients | 82.4% |
| Utility level in PFS | 0.8933 |
| Utility level in PPS | 0.8634 |
| One-off QALY decrement associated with AEs – Atezolizumab+Bev | -0.0016 |
| One-off QALY decrement associated with AEs – Sorafenib | -0.0014 |
| Time to off treatment – Distribution Atezolizumab | Exponential (rate = 0.0786) |
| Time to off treatment – Distribution Bevacizumab | Exponential (rate = 0.0852) |
| Time to off treatment – Distribution Sorafenib | Exponential (rate = 0.2003) |
| Progression-free survival – Distribution Atezolizumab+Bevacizumab | Exponential (rate = 0.0853) |
| Progression-free survival – Distribution Sorafenib | Exponential (rate = 0.1358) |
| Overall survival – Distribution Atezolizumab+Bevacizumab | Exponential (rate = 0.0348) |
| Overall survival – Distribution Sorafenib | Exponential (rate = 0.0512) |
| Overall survival with RWE adjustment [Guyot} – Distribution Sorafenib | Exponential (rate = 0.0486) |
| Cost per vial – Atezolizumab 1,200 mg | 3,569.52 € |
| Cost per vial – Bevacizumab 100 mg | 168.59 € |
| Cost per vial – Bevacizumab 400 mg | 620.40 € |
| Cost per pack – Sorafenib 112 tabs | 1,280.10 € |
| Vial sharing | No |
| Administration cost – IV GHM 28Z07Z | 431.08 € |
| Transportation cost | 52.42 € |
| Dispensation fees | 0.51 € |
| Packaging fees | 1.02 € |
| Weekly adverse event costs - Atezolizumab+Bevacizumab | 31.10 € |
| Weekly adverse event costs - Sorafenib | 41.65 € |
| Weekly supportive care cost – PFS Atezolizumab+Bevacizumab | 59.00 € |
| Weekly supportive care cost – PFS Sorafenib | 32.88 € |
| Weekly supportive care cost – PFS Atezolizumab+Bevacizumab | 32.88 € |
| Terminal care one-off cost | 6,357.38 € |
| % of patients that received a second line after Atezolizumab+Bev | 35.71% |
| % of patients that received a second line after Sorafenib | 52.12% |
| Total post discontinuation one-off cost after Atezolizumab+Bev | 4,189.05 € |
| Total post discontinuation one-off cost after Sorafenib | 11,803.69 € |
